# Effects of shear stress path and roughness on shear creep behavior of marine clay-concrete interface

**DOI:** 10.1038/s41598-023-37854-y

**Published:** 2023-07-01

**Authors:** Wangjing Yao, Tao Zhang, Qianshen Chen, Jiuchun Sun, Sifa Xu, Zhouxiang Ding, Zhe Wang

**Affiliations:** 1 Institute of Geotechnical Engineering, Zhejiang University of Technology, Hangzhou, China; 2 Tengda Construction Group Co. Ltd., Shanghai, China; 3 Department of Mechanical Engineering, University of Saskatchewan, Saskatoon, SK S7N 5A9 Canada

**Keywords:** Ocean sciences, Engineering, Civil engineering

## Abstract

Floating piles have been widely employed as foundations in coastal regions abounding with marine clay. A growing concern for these floating piles is their long-term performance of bearing capacity. To better understand the time-dependent mechanisms behind the bearing capacity, in this paper a series of shear creep tests was conducted to study the effects of load paths/steps and roughness on shear strain of the marine clay-concrete interface. Four main empirical features were observed from the experimental results. First, the creep process of the marine clay-concrete interface can be largely decomposed into the instantaneous creep stage, the attenuation creep stage and the uniform creep stage. Second, the creep stability time and the shear creep displacement generally increase as the shear stress level increases. Third, the shear displacement rises as the number of loading steps drops under the same shear stress. The fourth feature is that under the shear stress condition, the rougher the interface is, the smaller the shear displacement is. Besides, the load-unloading shear creep tests suggest that: (a) shear creep displacement typically contains both viscoelastic and viscoplastic deformation; and (b) the proportion of unrecoverable plastic deformation increases with increasing shear stress. These tests confirm that the Nishihara model can provide a well-defined description of the shear creep behavior of marine clay-concrete interfaces.

## Introduction

Over the past decades, long floating piles has been widely employed in offshore infrastructure around the world. The current trend indicates that a major concern emerges in the long-term performance of the marine clay-pile interface. This is because the marine clay surrounding the floating piles exhibits significant rheological properties and high sensitivity. These features differentiate the frictional behavior of the marine clay-concrete interface from that of pure clay. In the literature, the properties of marine clay have been found to considerably affect the long-term bearing capacity of pile foundations^1–4^. In addition, other key factors, such as the roughness of the interface and the shear stress path, also play a crucial part in the long-term mechanical properties of the soil–concrete interface. So far, however, these two vital factors have not been closely investigated within the context of long-run frictional behavior of the marine clay-concrete interface.

There is a growing body of publications that recognizes the significance of creep in engineering practice. For example, at the *First International Congress of Soil Mechanics and Foundation Engineering*, Buisuman^5^ argued that secondary consolidation of clay occurs after the end of primary consolidation without a full completion. Buisman's point was further confirmed by the later Vienna experiment, which lasted 42 years on clay samples. After that, numerous tests on rheological properties of soft soils have been carried out^6–8^. By comparison, the behavior of soil-pile interfaces is more complicated than that of homogeneous clay. This is because the surface of the pile with large modulus tends to constrain the soil around under the vertical working load. The mechanical properties of soil-pile interface are thus different from that of the soil and the pile^9,10^. As for the pile foundation of building structures, it mainly bears constant vertical load under normal conditions. Such constant vertical load gives rise to shear creep deformation of soils around the pile. The shear creep behavior of soil-structure interface, in turn, is arguably responsible for the long-term bearing capacity of pile foundation. This mechanism illustrates the significance of the current study.

Recent years have witnessed an encouraging progress of the shear creep behavior of soil-structure interfaces. These investigations have put the emphasis on such contributing factors as soil properties, moisture content, interface materials, temperature and normal stress levels. For example, Chen et al.^11^ examined the shear creep behavior of the soil-bolt interface and found that the creep displacement of the soil-bolt interface increased with increasing water content and decreasing dry density. Stavropoulou et al.^12^ utilized the claystone-concrete interface under normal stresses of 4 and 12 MPa to investigate the time-dependent behavior of the interface. In that research, the interface strain experienced a rate decay stage, then a uniform speed stage and finally an acceleration stage during the shear creep process. The dry and wet conditions of the concrete interface affected the development of the interface shear creep. He^13^ has studied the shear creep behavior of the interface with frozen soil-concrete under a maximum normal stress of 200 kPa at −1 °C, −2 °C, and −4 °C. The horizontal displacement of interface was observed to decrease with decreasing temperature. Yang et al.^14^ employed the clay-concrete interface to investigate the effects of roughness on time-dependent behavior of the interface. Six kinds of joint roughness coefficient (JRC) were addressed in those tests. Their results indicated that the long-term cohesion of the interface increases first and then decreases as the JRC increases. However, there are no obvious rules about the determination of friction angles in that study. For marine soft clay, Hu and Luo^9^ applied permeable stone to the simulation of pile interfaces, and examined the shear creep behavior on the pile-clay interface of Shanghai soft soil. This investigation showed that the shear creep displacement of pile-clay interface is smaller under the same shear load in comparison with that of clay. Although the shear creep behavior of soil-structure interface has been investigated as mentioned above, for marine soft clay, inconsistencies are noticed between the effects of interface roughness and horizontal load levels on shear creep behavior. As a consequence, the focus of this present study is on such effects.

Shear creep constitutive models have been developed for engineering applications. Based on shear creep tests, the shear creep behavior of pile-soil interface is generally described by time-dependent load–displacement curves^12–14^. Owing to the time dependence of the interface shear creep, a time-dependent shear creep constitutive model is required to describe the corresponding deformation characteristics. Using the concept of physical mechanics and mechanical analysis, a component model was established^15,16^. This model primarily calculated the relationship between shear deformation and time through the combination of components such as spring, dashpot and slider. The component models can be instantiated in the two-element Kelvin-Voigt model and Bingham model, the three-element generalized Kelvin model, the four-element Komamura-Huang model and Schiffman model, the five-element Nishihara model, and among others. Further, Zhang et al.^17^ conducted model tests on the creep behavior of the bolt-clay interface, analyzed the loading creep curve, and established a generalized Kelvin-Voigt model with five elements. To describe the accelerated creep stage in the shear creep process, Xiao^18^ connected the Nishihara model in parallel with the variable η of a Newtonian viscous cylinder and described the accelerated creep stage through the change of the η value. Yang et al.^14^ proposed a Nishihara model to account for instantaneous elastic modulus damage for clay-concrete interfaces with different interface roughness. However, He^13^ improved the η of the plastic dashpot and obtained the improved Nishihara model for the shear creep of frozen soil-structures interface, taking into account the hardening of soil and the damage of interface during the shear process. For marine clay-concrete interface, few previous studies have given sufficient consideration to the appropriate constitutive models that are essential to describe the shear creep behavior of concern.

The objective of this paper is to gain further understanding of the shear creep behavior of marine clay-pile interfaces using shear creep tests. In this work, the emphasis is on the relationship between time and horizontal displacement of the interface under fixed horizontal and normal loads. To achieve the objective, the present research is conducted from the following three aspects: (a) the proportion of the displacement in terms of the instantaneous creep stage, the attenuated creep stage and the uniform creep stage; (b) the effects of roughness and stress path on shear creep deformation; and (c) the shear deformation mechanisms based on multi-staged loading and unloading tests. Finally, a discussion is performed on the appropriate component model than can describe the shear creep behavior of the interface. The outcomes of the present experimental investigation complement those of prior studies.

## Test scheme

### Test materials

Fine-aggregate concrete and marine clay were used in this study to simulate the pile and soil, respectively. According to the C30 fine stone concrete standard (see the code for design concrete structures^19^), the concrete specimens were prepared by mixing water, cement, sand and fine stone in the ratio of 0.421: 1: 1.53: 1.59. After 28 days of maintenance, the specimens have a compressive strength of 34.8 MPa and a tensile strength of 3.58 MPa.

The marine clay in this study was acquired from the mud flat at Taizhou, Zhejiang Province, China. Soil samples were prepared according to the *Standard for soil test method*^20^.Saturated soil samples were made with the target moisture content of 20%. The soil samples were poured into a sampler equipped with a ring knife in three layers and then hammered 25 times each time. Saturation of the soil sample was accomplished by the vacuum pumping saturation method. A stacked saturator was selected, into which the prepared soil samples were sequentially filled. After the completion of vacuum pumping saturation of each specimen, the moisture content was maintained at 20%.

### Test equipment

**Figure 1:**
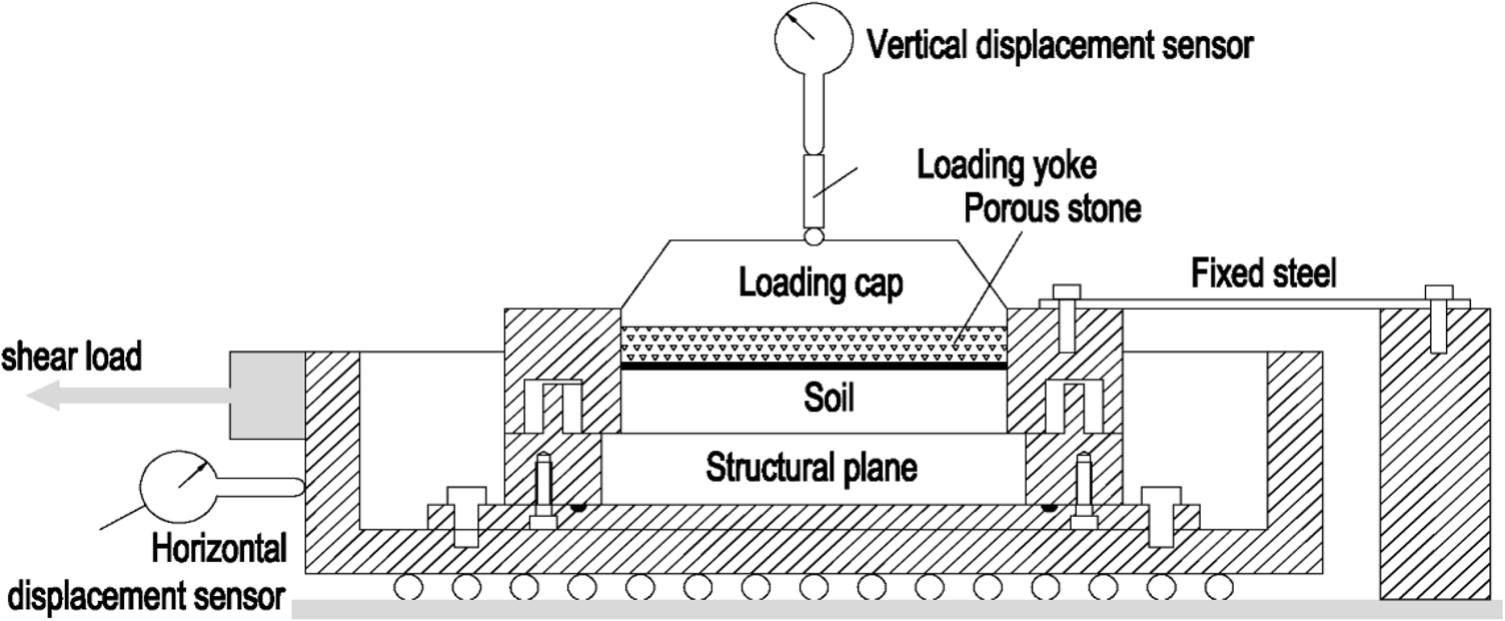
Schematic diagram of the shear creep system.

We modified a direct shear test system for the present testing, as illustrated in Fig. 1. Distinguished from the conventional test system, the re-designed system contains two modules for the horizontal load and the constant cross-sectional shear area. The precision of the displacement measuring device is 0.01 mm.

### Shearing method

The shearing testing was preceded with the consolidation of soil samples under the normal stresses (50, 100, 200, 300 kPa) in the shear creep system until the vertical deformation rate drops less than 0.005 mm/h. The horizontal shear load was gradually applied stepwise. For shear creep tests, the loading duration at different stress levels can be determined by the strain rate variation, as there is no standardized creep stability standard available. In the study of shear creep of soil, Sun^21^ pointed out that if the deformation is less than 0.01 mm within 10,000 s, then the creep can be regarded as reaching a stable level, thus the next stress level can be loaded.

### Test method

To study the influence of stress level and normal load on shear creep characteristics of soil-concrete interface, the following experimental design was carried out. Different normal load and shear load levels were set up according to the actual working conditions. The shear creep characteristics of interface under normal load and stress level were investigated.

First, direct shear tests of contact surfaces were performed to obtain the shear strength of soil-contact surfaces under different normal stress (100, 150, 200, 300 kPa). According to the results of the direct shear test, the experiment is classified into four groups, as shown in Table 1. The interface of the group 1 is rough, and the rest are smooth. Group 2 is the loading and unloading test. Group 3 and group 4 are five-level loading and three-level loading under 100 kPa normal stress respectively, while Group 5 is six-level loading under 150 kPa normal stress. Group 6 is six-level loading under 200 kPa normal stress. Group 7 and group 8 are nine-level loading and five-level loading, respectively.

**Table 1:** Sample conditions for shear creep test.

Normal stress (kPa)	Number	Shear stress levels						Shear strength (kPa)
100(rough)	1	15.8	23.7	31.6	39.5	47.4	55.3	
100	2	15.8	0	23.7	0	39.5	0	
100	3	15.8	23.7	31.6	39.5	47.4		55.14
100	4	15.8	31.6	47.4				
150	5	15.8	31.6	47.4	55.3	63.2	71.1	74.78
200	6	15.8	31.6	47.4	63.2	79	94.8	96.49
300	7	15.8	31.6	47.4	63.2	79	94.8	
		110.6	126.4	142.2				129.23
300	8	31.6	63.2	94.8	126.4	158		

The results of different normal stress tests may be coincident with past research. The results of different loading steps, different roughness and load-unload tests have some new points.

## Results and discussion

### Result for different normal stress

**Figure 2:**
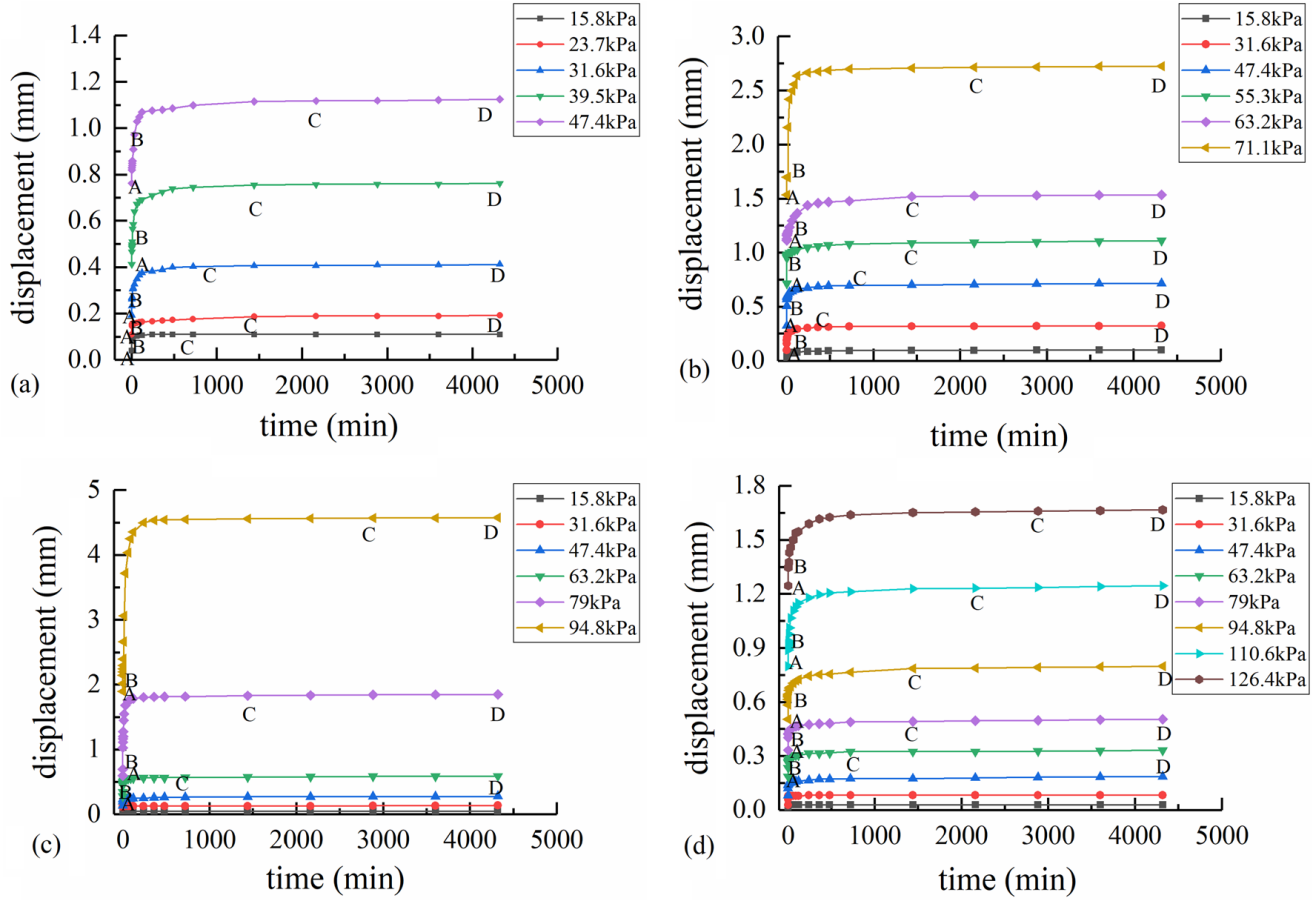
Time-horizontal displacement curves under different normal stresses: (**a**) 100 kPa normal stress; (**b**) 150 kPa normal stress; (**c**) 200 kPa normal stress; (**d**) 300 kPa normal stress.

The time-horizontal displacement curves under different normal stress are shown in Fig. 2a–d. In Fig. 2a–d, the curves are divided into section AB (instantaneous creep stage), Section BC (attenuated creep stage) and Section CD (uniform creep stage), based on strain rate calculated from time-horizontal displacement data. The proportion of different stages of shear strain under different shear stress is shown in Tables 2, 3, 4, 5, 6. The instantaneous creep stage means that the shear displacement at the moment when the shear stress is applied. The attenuated creep stage means that the shear displacement increases but the shear rate decreases. The uniform creep stage means that shear displacement increases at a constant rate. And the accelerated creep stage means that the shear displacement increases and the shear rate increases.

**Table 2:** The proportion of strain of each shear stress level under different normal stress.

Shear stress	Normal stress			
	The proportion of strain of each shear stress level (%)			
	100 kPa	150 kPa	200 kPa	300 kPa
15.8 kPa	9.8	3.67	1.1	1.79
23.7 kPa	7.38			
31.6 kPa	19.56	8.29	1.83	3.17
39.5 kPa	31.11			
47.4 kPa	32.15	14.33	3.01	6.23
55.3 kPa		14.59		
63.2 kPa		15.47	6.75	8.74
71.1 kPa		42.93		
79 kPa			27.49	10.36
94.8 kPa			59.82	17.6
110.6 kPa				26.78
126.4 kPa				25.33

**Table 3:** Group 2 test (normal 100 kPa) creep stage strain under different levels of load.

Shear creep stages	The proportion of strain under all stress levels (%)				
	15.8 kPa	23.7 kPa	31.6 kPa	39.5 kPa	47.4 kPa
Instantaneous creep deformation	63.64	44.58	18.18	17.14	10.54
Attenuation creep deformation	36.36	48.19	77.73	78.57	83.74
Uniform creep deformation	0.00	7.22	4.09	4.29	5.72
Accelerated creep	0	0	0	0	0

**Table 4:** Group 4 test (normal 150 kPa) creep stage strain under different levels of load.

Shear creep stages	The proportion of strain under all stress levels (%)					
	15.8 kPa	31.6 kPa	47.4 kPa	55.3 kPa	63.2 kPa	71.1 kPa
Instantaneous creep deformation	80	50	47	22.63	7.14	4.31
Attenuation creep deformation	20	37.45	43.24	66.84	83.34	87.57
Uniform creep deformation	0	12.55	9.76	10.53	9.52	8.12
Accelerated creep	0	0	0	0	0	0

**Table 5:** Group 5 test (normal 200 kPa) creep stage strain under different levels of load.

Shear creep stages	The proportion of strain under all stress levels (%)					
	15.8 kPa	31.6 kPa	47.4 kPa	63.2 kPa	79 kPa	94.8 kPa
Instantaneous creep deformation	60.00	48.20	37.06	28.06	14.32	7.30
Attenuation creep deformation	40.00	44.59	53.85	66.77	83.09	91.98
Uniform creep deformation	0.00	7.21	9.09	5.16	2.59	0.72
Accelerated creep	0.00	0.00	0.00	0.00	0.00	0.00

**Table 6:** Group 7 test (normal 300 kPa) creep stage strain under different levels of load.

Shear creep stages	The proportion of strain under all stress levels (%)				
	15.8 kPa	31.6 kPa	47.4	63.2 kPa	79 kPa
Instantaneous creep deformation	66.67	56.60	38.83	34.01	40.46
Attenuation creep deformation	33.33	43.40	56.41	61.22	50.81
Uniform creep deformation	0.00	0.00	4.76	4.77	1.73
Accelerated creep	0.00	0.00	0.00	0.00	0.00

Shear creep stages	94.8 kPa	110.6	126.4 kPa		
Instantaneous creep deformation	28.21	20.13	18.80		
Attenuation creep deformation	68.36	76.07	77.16		
Uniform creep deformation	3.43	3.80	4.04		
Accelerated creep	0.00	0.00	0.00		

In Fig. 2, the long-term shear strength of the interface increases with the increase of normal stress, while the horizontal displacement of the interface is similar at each shear stress level. The shear creep curve of the contact surface of marine soft soil-concrete generally includes the instantaneous creep stage, the attenuation creep stage and the uniform creep stage, which is consistent with He^13^ and Liu^22^. The instantaneous creep stage mainly involves the elastic deformation when the shear stress is applied. The attenuated creep stage occurs after the shear stress is applied. The horizontal displacement gradually increases with time, whereas the horizontal displacement rate gradually decreases and finally approaches the stable creep stage. In the steady creep stage, the displacement of the interface under constant load increases with time at a constant slow rate.

At the instant of the application of the shear stress (time *t* = 0), the instantaneous creep occurs at the interface. As shown in Fig. 2, at the instant of loading, creep curves at all stress levels are approximately vertical segments. The instantaneous creep deformation increases as shear stress level increases. For example, as shown in Fig. 2c, the shear stresses from the first stage to the sixth stage are 15.8, 31.6, 47.4, 63.2, 79, and 94.8 kPa, respectively. The instantaneous creep deformation from the first stage to the sixth stage (Section AB) is 0.03, 0.05, 0.07, 0.07, 0.09, and 0.12 mm, respectively. Furthermore, as the loading time continues, the stage of shear creep transfers from the instantaneous creep stage to the attenuated creep stage.

When the shear stress level is low, the shear creep has a short stability time (stable within 24 h). The shear creep primarily includes instantaneous creep deformation and attenuation creep deformation; however, the uniform creep deformation nearly does not exist.

Figure 2 and Table 3 through 6 indicate that when the normal stress is 100, 150, 200 and 300 kPa, the displacement generated by the interface under the first shear stress load are 0.11, 0.1, 0.05 and 0.03 mm, respectively. The proportion of the uniform creep (CD section) is 0%. The proportion of the instantaneous creep deformation (AB section) is 63.64%, 80%, 60%, and 66.67% respectively, and the rest is the attenuated creep deformation (BC section). In this state, the shape of the curve is mainly a small vertical segment at the beginning, and then it turns into an approximate right angle. It is because that an elastic and viscoelastic deformation occurs when it is subjected to a small constant stress. Then, under the condition of constant shear stress, the horizontal displacement rate of the interface decreases rapidly. The reason for this is that the bonded water viscosity between the concrete and the soil gradually plays a dominant role. Meanwhile, there is friction resistance between concrete and soil particles. The low shear stress is difficult to overcome the friction resistance, accordingly no displacement changes occurred in a long test time. If the load is removed at this moment, most of the horizontal displacement can be recovered. This was confirmed in subsequent experiments.

The horizontal displacement of the interface climbs steadily as the shear stress increases, and the shear creep deformation of the interface is principally the attenuated creep deformation. Figure 2c and Table 5 depict that the shear stresses from the first stage to the sixth stage are 15.8, 31.6, 47.4, 63.2, 79, and 94.8 kPa, respectively. The horizontal displacement from the first stage to the sixth stage is 0.05, 0.08, 0.14, 0.31, 1.26, and 2.74 mm, respectively. The morphology of the curve gradually changes from a vertical straight section to a curve of different radians, which indicates that the shear creep deformation enters the attenuated creep stage. The proportion of attenuated creep deformation of the interface is 40%, 44.59%, 53.85%, 66.77%, 83.09%, and 91.98%, respectively. The deformation value of the attenuated creep rises steadily with the increase of shear stress, and the proportion of instantaneous creep deformation decreases gradually. At the same time, the duration of attenuated creep stage increases as shear stress increases. For example, when the normal stress is 200 kPa and the shear stress is 79 kPa and 94.8 kPa, respectively, the duration of the attenuated creep deformation about 1500 min and 2880 min. Friction resistance of interface increases, owing to free water and bound water in the soil sample are slowly discharged and the soil particles are compacted with each other as time increases. In practice, many large bridges and airports built on soft soil foundations or even in coastal soft soil areas will also experience a long creep deformation process. In this process, the creep deformation rate keeps a trend of slow decline, but the settlement will continue to increase. As a consequence, the foundation soil and pile foundation will produce a large cumulative settlement for years and months under the action of the constant load of the superstructure. This effect will bring potential safety risks to the pile foundation project.

After attenuated creep stage, the shear creep deformation changes to uniform creep. Shear creep deformation rate is stable as the friction resistance reaches ultimate value. Due to the limitation of test conditions, it is considered to have entered a stable stage when the shear creep deformation rate is less than 0.005 mm/d. In the constant creep stage, the interface continuously generates displacement without stopping under constant shear stress^23^.

However, when the shear stress exceeds the long-term shear strength of the interface, the shear creep deformation increases rapidly (more than 3 mm) in a short time (about 30–60 min), and finally failed. Since the deformation rate is employed as a stable index rather than a time scale in this test, no accelerated creep phase occurs during the test. This result accords with the result of Fan^24^.

### Result for different loading steps

**Figure 3:**
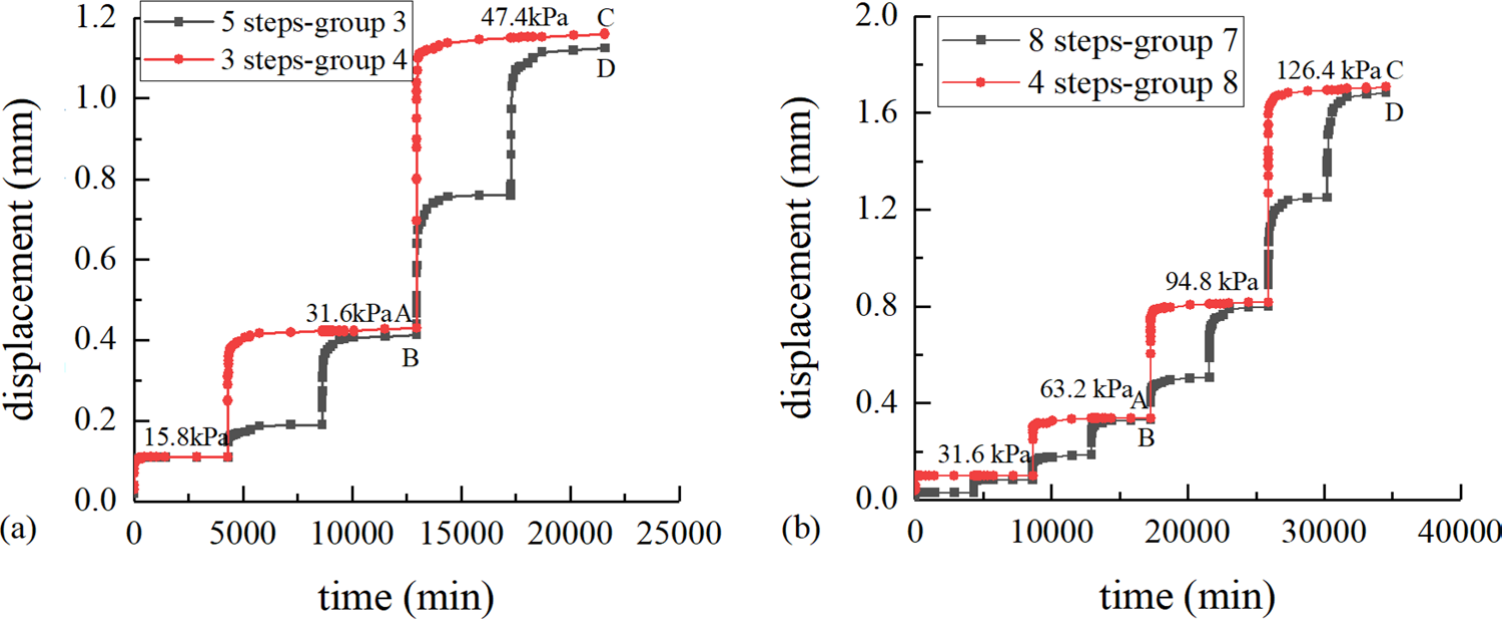
Time-horizontal displacement curves for different loading steps: (**a**) 100 kPa normal stress; (**b**) 300 kPa normal stress.

The shear creep behavior is affected by the stress path. The time-horizontal displacement curves about different loading steps under 100 kPa normal stress are shown in Fig. 3a. Group 3 used a 5-step load, while group 4 used a 3-step load (Table 1). The time-horizontal displacement curves about different loading steps under 300 kPa normal stress are shown in Fig. 3b. Group 7 used an 8-step load, while group 8 used a 4-step load (Table 1).

Compared with group 3, the horizontal displacement of group 4 is increased by 0.017 mm(A-B) under 31.6 kPa shear stress and 0.035 mm(C-D) under 47.4 kPa shear stress. By comparison with group 7, the horizontal displacement of group 8 is increased by 0.004 mm(A-B) under 63.2 kPa shear stress and 0.024 mm (C-D) under 126.4 kPa shear stress. It indicates that the smaller the number of loading steps, the larger the ultimate shear creep deformation under the same ultimate shear stress. The horizontal displacement difference of interface under low normal stress appears to be larger than that under high normal stress.

### Result for different roughness of interface

**Figure 4:**
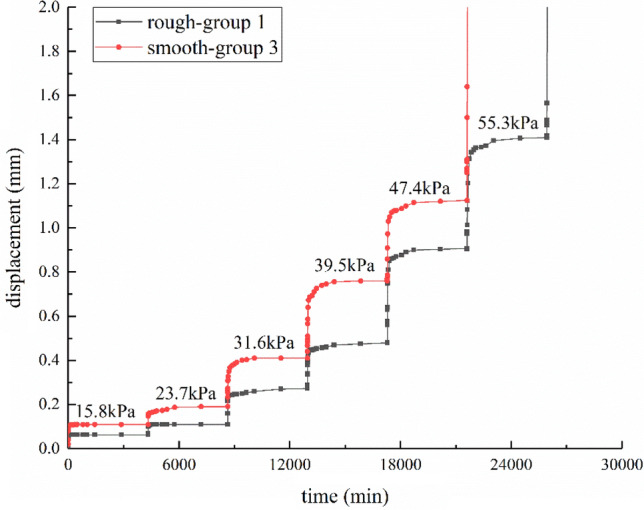
Time-horizontal displacement curves for different interface roughness under 100 kPa normal stress.

**Figure 5:**
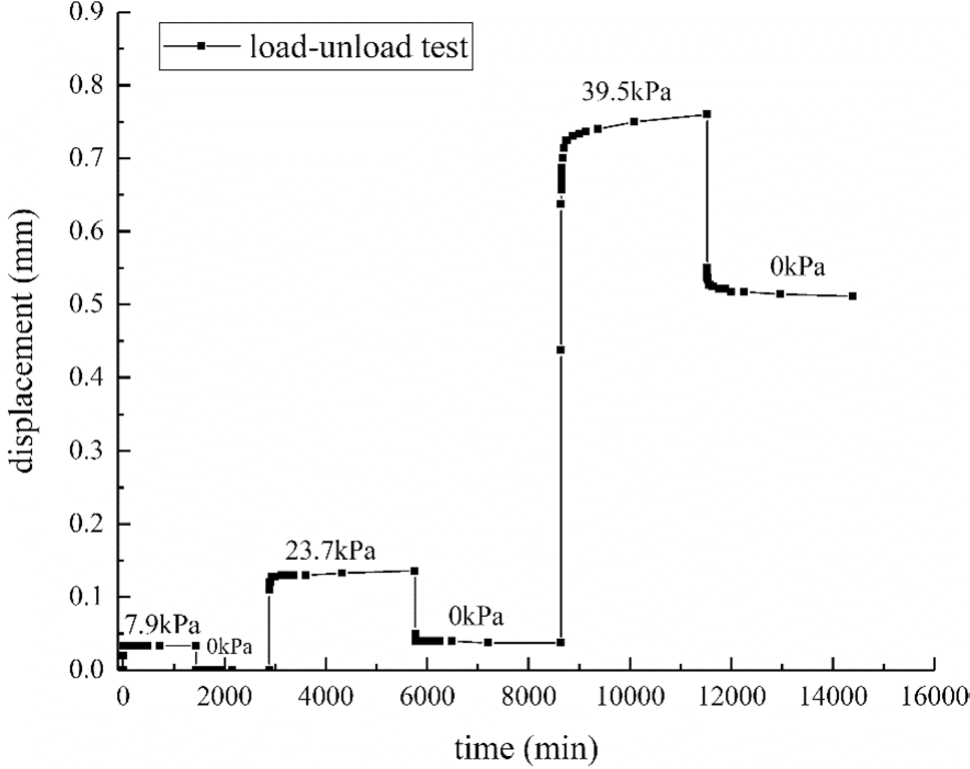
Time-horizontal displacement curves for loading–unloading under 100 kPa normal stress.

The time-horizontal displacement curves about different interface roughness under 100 kPa normal stress are shown in Fig. 4. Group 1 is rough interface, while group 2 is smooth interface (Table 1). Rough interface is uniformly inlaid with fine stones with a diameter of about 1 mm. As illustrated in Fig. 5, under the same load steps (15.8, 23.7, 31.6, 39.5, 47.4, and 55.3 kPa), the horizontal displacement of rough interface is 0.063, 0.11, 0.249, 0.45, 0.903, and 1.149 mm, and the horizontal displacement of smooth interface is 0.11, 0.19, 0.413, 0.763, and 1.125 mm. By comparison with group 1, the horizontal displacement of group 2 is increased by 1.75, 1.73, 1.66, 1.70, and 1.25 times, respectively. As observed from the curve shape, with the increase of shear stress, the creep curve of smooth interface has a circular arc turning point while the creep curve of rough interface is closer to a right angle. Either in terms of the instantaneous shear creep or the subsequent shear creep with time, group 2 is noticeably higher than group 1. The results of the horizontal displacement with different roughness in this paper are consistent with Yang et al.^14^.

### Result for load-unload test

Since the elastic deformation is recoverable, the elastic deformation of the interface can be recovered after the shear stress is removed. To clarify the situation of the elastic deformation and plastic deformation of the sample in the loading process, a loading–unloading test was carried out.

The time-horizontal displacement curves about loading–unloading under 100 kPa normal stress are shown in Fig. 5. The shear stress steps are 7.9, 0, 23.7, 0, 39, and 0 kPa.

At the moment when the shear stress is applied, the interface will produce instantaneous elastic deformation, and the value of instantaneous elastic deformation increases as shear stress increases.

When the stress level is low, the deformation can be completely recovered and most of it is instantaneous rebound. When the stress level is high, such as the second stage load of 23.7 kPa, the instantaneous creep deformation generated is 0.11 mm, and the total creep deformation is 0.137 mm. When all the loads are removed, the instantaneous rebound is 0.087 mm, which is about 80% of the instantaneous creep deformation. After that, it is slowly rebounded to approximately 0.013 mm within 36 h, yet the remaining 0.037 mm cannot be recovered. It indicates that the shear creep deformation changes from recoverable deformation to unrecoverable deformation as the shear stress increases. Besides, the proportion of elastic deformation decreases with the increase of shear stress.

For the third stage load of 39.5 kPa, the instantaneous creep deformation generated is 0.437 mm, and the total creep deformation is 0.76 mm. When all the loads are removed, the instantaneous rebound is 0.21 mm, which is about 48.05% of the instantaneous creep deformation. After that, it is slowly rebounded to about 0.039 mm within 36 h, and the remaining 0.511 mm cannot be recovered. The instantaneous creep deformation includes not only elastic deformation but also plastic deformation. Compared with the second stage, the viscoelastic deformation and the viscoplastic deformation increase as the shear stress increases.

## Creep model

**Figure 6:**
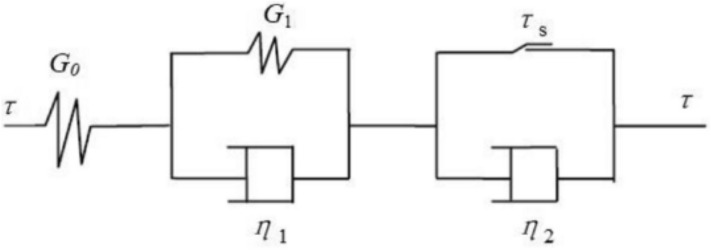
Nishihara model.

The shear creep deformation includes elastic deformation, viscoelastic deformation, plastic deformation, and visco-plastic deformation. Nishihara model (Fig. 6) is deemed ideal for simulating the elastic, viscoelastic and visco-plastic deformation. The creep equation of the Nishihara model can be expressed as:

1 $$ \gamma = \gamma^{e} + \gamma^{ve} + \gamma^{vp} = \left\{ {\begin{array}{*{20}l} {\frac{\tau }{{G_{0} }} + \frac{\tau }{{G_{1} }}\left( {1 - e^{{ - \frac{{G_{1} }}{{\eta_{1} }}t}} } \right)} \hfill & {\tau < \tau_{s} } \hfill \\ {\frac{\tau }{{G_{0} }} + \frac{\tau }{{G_{1} }}\left( {1 - e^{{ - \frac{{G_{1} }}{{\eta_{1} }}t}} } \right) + \frac{{\tau - \tau_{s} }}{{\eta_{2} }}t} \hfill & {\tau \ge \tau_{s} } \hfill \\ \end{array} } \right. $$

where $\gamma$ is the total strain; ${\gamma }^{e}$ is the strain of Hooke body; ${\gamma }^{ve}$ is strain of the viscoelastic body; ${\gamma }^{vp}$ is the strain of viscoplastic body; τ is the shear stress; ${G}_{0}$ is the shear modulus of Hooke body;${G}_{1}$ is the shear modulus of viscoelastic body; ${\eta }_{1}$ is the viscosity coefficient of viscoelastic body;${\eta }_{2}$ is the viscosity coefficient of viscoplastic body; and ${\tau }_{s}$ is the yield stress.

By the fitting method, a global optimization algorithm was used to obtain the ${G}_{0}$, ${G}_{1}$, ${\eta }_{1}$, ${\eta }_{2}$ from the curve shown in Figs. 2a, c, and d. The results are listed in Tables 7, 8, 9, respectively.

**Table 7:** Parameters of Nishihara model under 100 kPa normal load.

Shear stress(kPa)	${G}_{0}$	${G}_{1}$	${\eta }_{1}$	${\eta }_{2}$
15.8	694.81	192.92	5.20E + 05	–
23.7	155.88	970.91	2.23E + 05	3.05E + 06
31.6	121.11	245.48	1.06E + 04	3.18E + 06
39.5	84.85	158.77	4.03E + 03	1.98E + 06
47.4	64.11	140.89	3.56E + 03	2.63E + 06

**Table 8:** Parameters of Nishihara model under 200 kPa normal load.

Shear stress(kPa)	${G}_{0}$	${G}_{1}$	${\eta }_{1}$	${\eta }_{2}$
15.8	263.67	1436.36	2.44E + 02	–
31.6	210.56	1755.56	6.91E + 03	–
47.4	203.39	1478.94	2.43E + 05	3.59E + 06
63.2	170.46	345.11	9.71E + 03	2.85E + 06
79	88.60	88.72	8.44E + 03	2.30E + 06
94.8	50.05	36.85	1.02E + 03	1.83E + 06

**Table 9:** Parameters of Nishihara model under 300 kPa normal load.

Shear stress(kPa)	${G}_{0}$	${G}_{1}$	${\eta }_{1}$	${\eta }_{2}$
31.6	277.19	1227.66	5.99E + 02	–
47.4	249.18	1649.27	1.55E + 04	–
63.2	203.35	1851.20	1.74E + 04	3.01E + 06
79	165.09	1349.97	1.26E + 04	4.63E + 06
94.8	146.95	542.55	5.32E + 03	5.92E + 06
110.6	101.85	392.30	3.58E + 03	6.22E + 06
126.4	85.03	448.43	4.40E + 03	8.71E + 06

From the fitting results, the Nishihara model can satisfactorily describe the process of attenuation shear creep and uniform shear creep of marine clay-concrete interface, with the R square above 0.94. This proves that the calculated results are in good agreement with the experimental values.

As shown in Tables 7, 8, and 9, the shear modulus of Hooke body ${G}_{0}$ decreases as the shear stress τ increases. It suggests that the greater the shear stress, the easier the interface deformation. The shear modulus of viscoelastic body ${G}_{1}$ also decreases as the shear stress τ increases.

## Conclusion

Using the modified shear creep system, this paper conducted shear creep tests to investigate the shear creep behavior of marine clay-concrete interface and the effect of load steps and roughness on shear creep behavior of marine clay-concrete interface. The following conclusions may be summarized:

1. The creep process of marine clay-concrete interface largely contains the instantaneous creep stage, the attenuation creep stage and the uniform creep stage. The creep stability time and the shear creep displacement increase as shear stress level increases.
2. The shear creep deformation of the interface is also related to the loading conditions and the roughness of the interface. The smaller the number of loading steps, the larger the ultimate shear creep displacement under the same ultimate shear stress. Under the condition of horizontal stress, the shear displacement decreases as the roughness of interface increases.
3. Continuous load-unloading shear creep tests show that the shear creep displacement includes viscoelastic and viscoplastic deformation. The Nishimoto model can characterize the viscoelasticity and viscoplasticity of shear creep of marine clay-concrete interface. This model can also describe the instantaneous creep stage, the attenuation creep stage, and the uniform creep stage for the testing in this paper.

## Data Availability

All data, models generated or used during this study are available from the corresponding author by request.
